# The PRL2 phosphatase up-regulates miR-21 through activation of the JAK2/STAT3 pathway to down-regulate the PTEN tumor suppressor

**DOI:** 10.1042/BCJ20240626

**Published:** 2025-04-11

**Authors:** Qinglin Li, Yunpeng Bai, Sarah M. Cavender, Yiming Miao, Frederick Nguele Meke, Emily L. Lasse-Opsahl, Peipei Zhu, Gina M. Doody, W. Andy Tao, Zhong-Yin Zhang

**Affiliations:** 1 Borch Department of Medicinal Chemistry and Molecular Pharmacology, Purdue University, 720 Clinic Drive, West Lafayette, IN 47907, U.S.A; 2 Department of Biochemistry, Purdue University, 720 Clinic Drive, West Lafayette, IN 47907, U.S.A; 3 Division of Haematology and Immunology, Leeds Institute of Medical Research, University of Leeds, U.K.; 4 The James Tarpo Jr. and Margaret Tarpo Department of Chemistry, Purdue University, 720 Clinic Drive, West Lafayette, IN 47907, U.S.A; 5 Purdue Institute for Cancer Research, Purdue University, 720 Clinic Drive, West Lafayette, IN, 47907, U.S.A; 6 Purdue Institute for Drug Discovery, Purdue University, 720 Clinic Drive, West Lafayette, IN, 47907, U.S.A

**Keywords:** JAK2, miR-21, PRL2, PTEN, STAT3

## Abstract

The phosphatases of regenerating liver (PRLs) are members of the protein tyrosine phosphatase (PTP) superfamily that play pro-oncogenic roles in cell proliferation, migration, and survival. We previously demonstrated that PRLs can post-translationally down-regulate PTEN, a tumor suppressor frequently inactivated in human cancers, by dephosphorylating PTEN at Tyr336, which promotes the NEDD4-mediated PTEN ubiquitination and proteasomal degradation. Here, we report that PRLs can also reduce PTEN expression by up-regulating microRNA-21 (miR-21), which is one of the most frequently overexpressed miRNAs in solid tumors. We observe a broad correlation between PRL and miR-21 levels in multiple human cancers. Mechanistically, PRL2, the most abundant and ubiquitously expressed PRL family member, promotes the JAK2/STAT3 pathway-mediated miR-21 expression by directly dephosphorylating JAK2 at Tyr570. Finally, we confirm that the PRL2-mediated miR-21 expression contributes to its oncogenic potential in breast cancer cells. Our study defines a new functional role of PRL2 in PTEN regulation through a miR-21-dependent post-transcriptional mechanism, in addition to our previously reported NEDD4-dependent post-translational PTEN regulation. Together, these studies further establish the PRLs as negative regulators of PTEN.

## Introduction

Protein tyrosine phosphorylation plays a crucial role in regulating signaling pathways that control cellular growth, proliferation, metabolism, migration, survival, and the immune response [1,2]. Proper levels of cellular protein tyrosine phosphorylation, maintained by the reversible and co-ordinated actions of protein tyrosine phosphatases (PTPs) and protein tyrosine kinases, are critical to maintaining a healthy cellular homeostasis [3]. The PTPs constitute a family of more than 100 members in humans [4]. Aberrant PTP activities are associated with various human diseases, including cancer, diabetes, and autoimmune dysfunction [5]. As part of the PTP superfamily, the phosphatases of regenerating liver (PRLs) comprise three members (PRL1, 2, and 3, encoded by *PTP4A1*, *2,* and *3*) that share over 75% of their amino acid sequence identity [6,7]. Overexpression of PRLs has been linked to cell proliferation, metastasis, and invasion through activation of several signaling pathways, including the Rho family of small GTPases, Src, STAT3, ERK1/2, and PI3K/AKT [3,8]. PRLs are also reported to control intracellular magnesium concentration through their association with the cyclin and cystathionine-β-synthase (CBS) domain divalent metal cation transport mediator (CNNM) magnesium transport regulator proteins [9]. However, the exact biochemical mechanisms underlying PRLs’ ability to promote tumor progression are not fully understood.

Among the three PRLs, PRL2 is the most abundant and ubiquitously expressed in both humans and mice [9–11]. Deletion of PRL2 causes placenta dysfunction, impairment of spermatogenesis, and hematopoietic insufficiency in mice due to an increased PTEN level and reduced PI3K/AKT signaling [12–15]. These observations suggest that PRL2 may down-regulate PTEN, thereby activating the PI3K/AKT pathway. PRL1 and PRL2 are functionally redundant in the context of PTEN regulation during spermatogenesis [11]. Overexpression of PRL3 in DLD-1 colon cancer cells has also been shown to enforce cell survival and oncogenesis by down-regulating PTEN and activating AKT [16]. Mechanistically, we uncovered that PRL2 can post-translationally down-regulate PTEN, a tumor suppressor frequently inactivated in human cancers [17,18], by dephosphorylating PTEN at Tyr336, thereby promoting NEDD4-mediated PTEN ubiquitination and proteasomal degradation [19]. This mechanism may apply to all PRLs since, in addition to PRL2, PRL1 and PRL3 can also promote PTEN polyubiquitination and degradation by catalyzing PTEN Tyr336 dephosphorylation [19]. Given the strong cancer susceptibility associated with subtle reductions in PTEN [20–22], the ability of PRL2 to reduce PTEN level provides a biochemical basis for its oncogenic propensity. Consequently, targeting PRL2 could provide a novel therapeutic strategy to restore PTEN function, thereby obliterating PTEN deficiency-induced malignancies. Indeed, we have shown that the removal of PRL2 elevates PTEN and improves the overall survival in PTEN heterozygosity-induced acute myeloid leukemia and cancer models [19,23]. Moreover, we also demonstrated that PRL2 deletion in p53 deficient mice attenuates tumor growth through up-regulation of PTEN [24]. These studies further solidify the mechanism by which PRL2 promotes tumorigenesis via the down-regulation of PTEN and establish PTEN restoration by PRL2 inhibition as a therapeutic approach for cancer treatment in both PTEN deficient and wildtype (WT) backgrounds.

Given the fact that the tumor suppressor function of PTEN can be compromised through a variety of mechanisms including genetic mutation, epigenetic silencing, post-transcriptional regulation, and post-translational modifications [25,26], we investigated whether the PRLs might also negatively regulate PTEN through other mechanisms beyond post-translational regulation [19]. MicroRNAs (miRNAs) are a class of naturally occurring small non-coding RNAs with a size of 19–25 nucleotides after maturation [27]. miRNAs can base pair with the three prime untranslated regions (3′-UTR) of messenger RNAs (mRNA) of target genes to control biological processes such as differentiation, proliferation, and apoptosis [28–30]. Ample evidence has established that some miRNAs, known as oncogenic miRNAs (oncomirs), are dysregulated and play critical roles in tumorigenesis and cancer progression [31–33]. Among these oncomirs, miR-21 is one of the most frequently overexpressed miRNAs in solid tumors [34]. Interestingly, most of the targets of miR-21 are tumor suppressors, including PTEN [35].

miR-21 is transcriptionally regulated by the signal transducer and activator of transcription 3 (STAT3) via the Janus kinase 2 (JAK2)/STAT3 pathway, which is essential for cell growth, differentiation, and immune responses [36–39]. Upon binding of growth factors or cytokines, such as IL-6, erythropoietin, leptin, and IFN-γ, to the extracellular domain of their specific cellular receptors, JAK2 is recruited to the dimerized receptor molecules, resulting in autophosphorylation of JAK2 on tyrosine residues [40,41]. JAK2 autophosphorylation elevates its kinase activity, thereby enabling the phosphorylation of the intracellular domains of the receptors, which creates docking sites for SH2 domain-containing proteins, including STAT3. STAT3 is then phosphorylated by JAK2 on Tyr705, leading to STAT3 dimerization and nuclear translocation to transcribe its target genes, including miR-21 [40]. It has been shown that the phosphorylation of JAK2 on Tyr221 increases its kinase activity, while the phosphorylation of JAK2 on Tyr570 inhibits its kinase activity, contributing to the rapid termination of ligand-mediated activation of JAK2 [41,42]. Interestingly, JAK2 Tyr570 is evolutionarily conserved but not found in JAK1, JAK3, or TYK2, suggesting a unique regulatory role of Tyr570 phosphorylation in JAK2 [41]. We previously found that PRL3 expression elevates STAT3 phosphorylation on Tyr705, which contributes to PRL3’s ability to promote cell proliferation, migration, and invasion [43,44]. It has also been found that the overexpression of PRL3 in colon cancer cells induced the expression of miR-21, miR-17, and miR-19a as a result of STAT3 activation [45]. Unfortunately, the mechanism for how PRL promotes STAT3 Tyr705 phosphorylation is unknown. Since PRL2 is capable of down-regulating PTEN, we speculated that PRL2 may also dephosphorylate JAK2 at Tyr570 to sustain JAK2/STAT3 activation, leading to increased miR-21 expression and reduced PTEN expression, through a post-transcriptional mechanism.

In the present study, we determined that miR-21 levels can be up-regulated by all members of the PRL family. We also found that miR-21 levels are positively correlated with the mRNA levels of at least one PRL family member in multiple human cancers. Our findings showed that PRL2, the most abundant and ubiquitously expressed PRL family member, dephosphorylates JAK2 at Tyr570 to activate the JAK2/STAT3 signaling pathway, leading to an increase in the transcription of miR-21, which down-regulates PTEN by targeting the 3′-UTR of the mRNA for PTEN. Furthermore, we demonstrated that the overexpression of PRL2 promotes breast cancer oncogenesis by up-regulating miR-21 and down-regulating PTEN. Our data provide a novel post-transcriptional mechanism for PRL2-mediated down-regulation of PTEN through the JAK2/STAT3/miR-21 pathway. Together with the previously established post-translational mechanism for PTEN down-regulation through PTEN Tyr336 dephosphorylation, our data further solidified the role of PRL2 as a negative regulator of PTEN, supporting the PTEN augmentation strategy for cancer treatment through targeting PRL2.

## Results

### PRLs promote miR-21 expression

**Figure 1:**
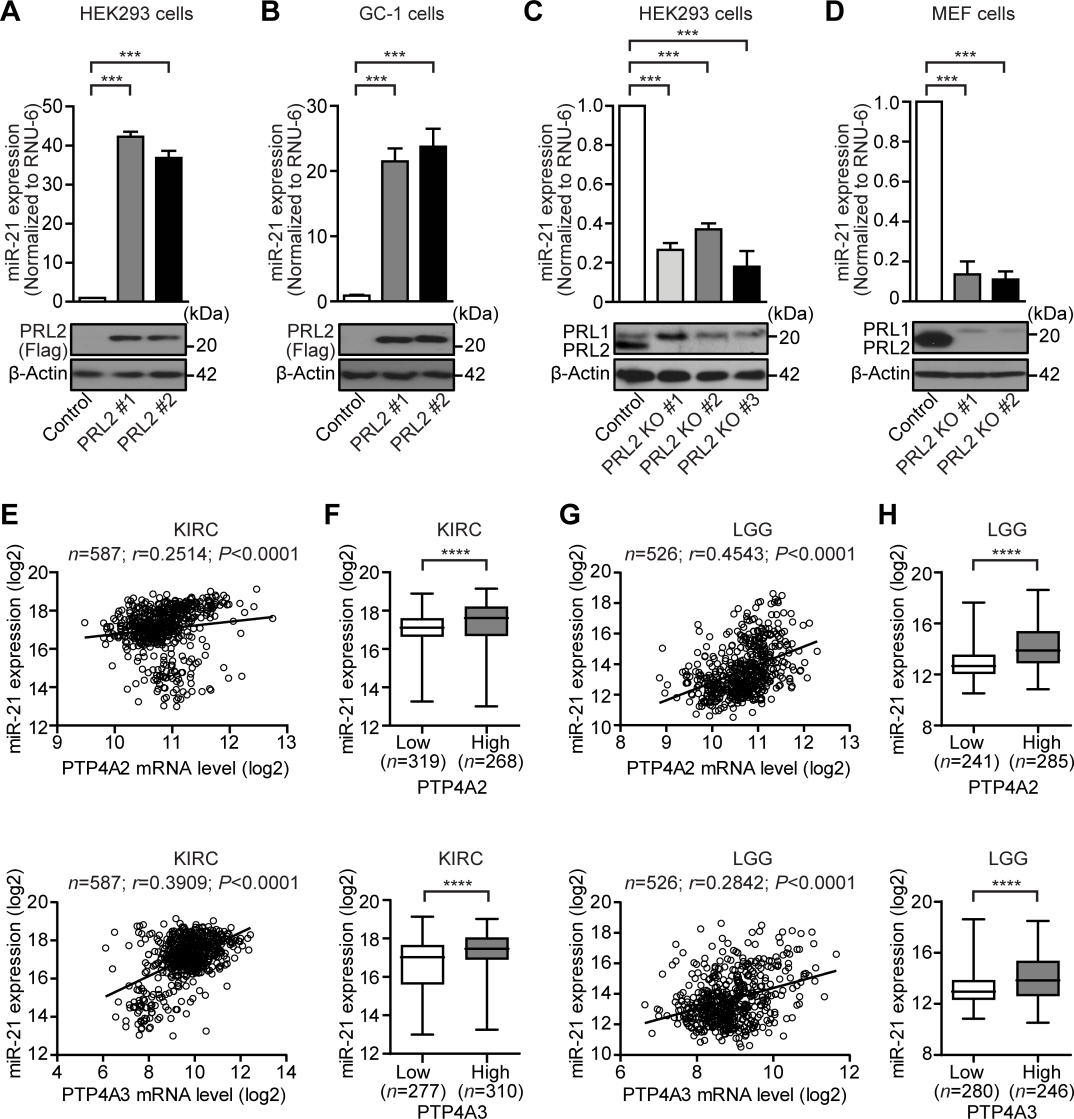
The PRLs promote miR-21 expression. (**A**) qRT-PCR analysis of miR-21 expression in HEK293 control and PRL2 overexpressing cells, normalized to RNU-6 snRNA. Each sample was analyzed in triplicate, and the Student’s t-test was used to measure significance (***P < 0.001). Western blotting showing PRL2 protein expression in PRL2-Flag or control vector transfected HEK293 cells. (**B**) qPCR analysis of miR-21 expression in GC-1 control and PRL2 overexpressing cells, normalized to RNU-6 snRNA. Each sample was analyzed in triplicate, and the Student’s t-test was used to measure significance (***P < 0.001). Western blotting was used to examine PRL2 expression in control and PRL2 overexpressing GC-1 cells. (**C**) qRT-PCR analysis of miR-21 expression in control and CRISPR-based PRL2 KO HEK293 cells, normalized to RNU-6 snRNA. Each sample was analyzed in triplicate, and the Student’s t-test was used to measure significance (***P < 0.001). Western blotting showing PRL2 protein expression in control and PRL2 KO HEK293 cells. (**D**) qPCR analysis of mouse miR-21 expression in control and PRL2 knockout MEF cells normalized to RNU-6 snRNA. Each sample was analyzed in triplicate, and the Student’s t-test was used to measure significance (***P < 0.001). Western blotting was used to examine PRL2 expression in control and PRL2 knockout MEF cells. (**E**) The mRNA level of PTP4A2 (top panel) or PTP4A3 (bottom panel) and miR-21 from Kidney renal clear cell carcinoma (KIRC, *n* = 587) were plotted and the Pearson rank correlation analyses were performed. Positive correlation coefficients suggest a positive correlation between PTP4A2 or PTP4A3 mRNA and miR-21 in the cancer samples (****P < 0.0001). (**F**) Patient samples from KIRC were divided into low-expression and high-expression groups based on the mean PTP4A2 (top panel) or PTP4A3 (bottom panel) mRNA level, and then the miR-21 level of the two groups was plotted. miR-21 level was significantly higher in the high-expression groups than the low-expression groups by Mann-Whitney tests (****P < 0.0001). (**G**) The mRNA level of PTP4A2 (top panel) or PTP4A3 (bottom panel) and miR-21 from brain low-grade gliomas (LGG, *n* = 526) were plotted and the Pearson rank correlation analyses were performed. Positive correlation coefficients suggest a positive correlation between PTP4A2 or PTP4A3 mRNA and miR-21 in the cancer samples (****P < 0.0001). (**H**) Patient samples from LGG were divided into low-expression and high-expression groups based on the mean PTP4A2 (top panel) or PTP4A3 (bottom panel) mRNA level, and then the miR-21 level of the two groups was plotted. The miR-21 level was significantly higher in the high-expression groups than in the low-expression groups by Mann-Whitney tests (****P < 0.0001).

Increasing evidence has demonstrated that miR-21 is consistently up-regulated in cancers [29,46], while overexpression of PRLs has also been linked to cancer metastasis and poor prognosis in various cancer types [3,47]. Since it was previously shown that PRL3 overexpression induces miR-21 up-regulation in colon cancer cells [45], we hypothesized that miR-21 expression might be augmented by all members of the PRL family. Indeed, we found that overexpression of PRL1, PRL2, or PRL3 could all increase miR-21 expression as measured by real-time quantitative PCR, with PRL2 displaying the highest capacity over PRL1 and PRL3 to induce miR-21 expression in human embryonic kidney HEK293 cells and mouse spermatogonia GC-1 cells (Supplementary Figure S1A,B). These observations suggest that PRLs can promote miR-21 expression in both human and mouse cells. Specifically, the level of miR-21 expression was about 40- and 21-fold higher in two independent PRL2 overexpressing HEK293 clones and GC-1 clones as compared with their control cells, respectively (Figure 1A,B). To determine whether the loss of PRL2 attenuates miR-21 expression, we generated PRL2-deleted HEK293 cell lines by using the clustered regularly interspaced short palindromic repeats (CRISPR)/Cas9 technology. We found that PRL2 deficiency decreases miR-21 expression by at least 60% in three independent PRL2 KO clones as compared with the control cells (Figure 1C). Similar results were obtained from siRNA-mediated PRL2 knockdown in HEK293 cells, although the effect was more moderate, possibly due to the transient nature of PRL2 siRNA expression (Supplementary Figure S1C). Furthermore, the miR-21 expression was more than 80% lower in PRL2 KO mouse embryonic fibroblast (MEF) cells as compared with the WT control cells (Figure 1D). Importantly, we found that, unlike the WT PRL2, the catalytically inactive PRL2-CSDA mutant (where the active site Cys101 and Asp69 were replaced by Ser and Ala, respectively) [19] was unable to alter miR-21 expression (Supplementary Figure S1D,E), indicating that the phosphatase activity of PRL2 is required for its ability to modulate miR-21 expression.

To further expand this finding to a more clinically relevant setting, we surveyed The Cancer Genome Atlas (TCGA) database to determine whether the up-regulation of PRLs coincides with an increase in miR-21 levels in human cancer samples. We noticed that the mRNA expression levels of PRL family members were positively correlated with miR-21 levels in a majority of the cancer types (Supplementary Figure S1F). More specifically, we found that the mRNA expression levels of both PRL2 and PRL3 were positively correlated with miR-21 expression in kidney renal clear cell carcinoma (KIRC) and brain low-grade gliomas (LGGs) (Figure 1E,G), with PRL2 (*r* = 0.2514) showing a weaker correlation than PRL3 (*r* = 0.3909) in KIRC while PRL3 (*r* = 0.2842) exhibiting a weaker correlation than PRL2 (*r* = 0.4543) in LGG. Importantly, patient samples with elevated PRL2 or PRL3 expression also showed higher miR-21 expression compared with low-expression groups (Figure 1F,H). Consistent with the finding in mouse spermatogonia GC-1 cells, a positive correlation was also obtained for PRL2 and miR-21 expressions in testicular germ cell tumors (TGCTs) (Supplementary Figure S1G,H). Most importantly, high expression of both PRL2 and PRL3 predicts worse survival in KIRC, while PRL2 high expression also predicts worse survival in LGG (Supplementary Figure S1I,J). Consistent with the PRL-miR-21 correlation, we also found that miR-21 high expression also predicts worse survival in both KIRC and LGG patients (Supplementary Figure S1I,J), suggesting that the positive correlation between PRL and miR-21 expression contributes to the poor prognosis in cancer patients. Considering that PRL2 exerted the strongest effect on miR-21 gene expression, we selected PRL2 for further analysis.

### PRL2-mediated miR-21 expression contributes to its oncogenic property

**Figure 2:**
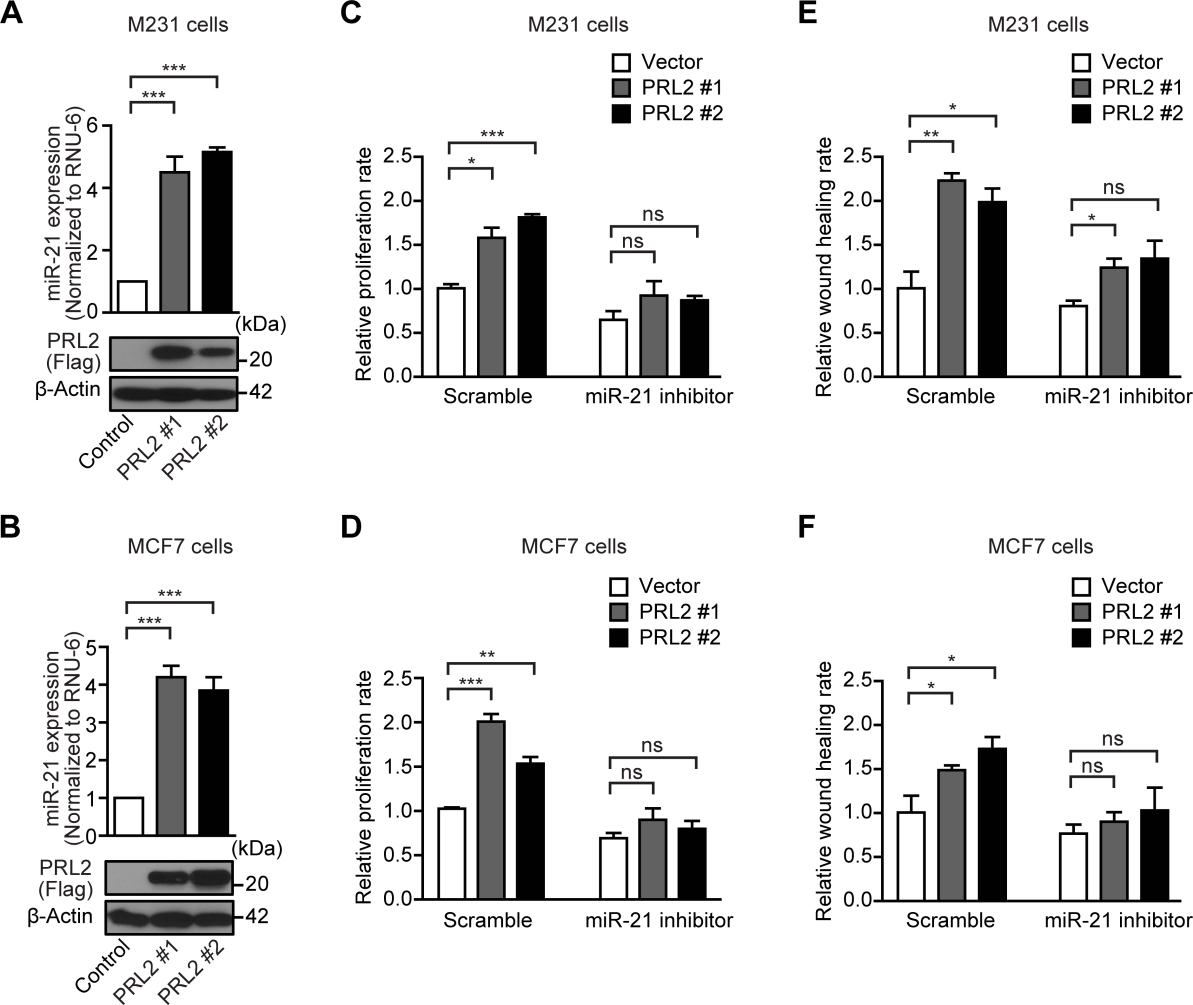
PRL2-mediated miR-21 expression contributes to its oncogenic function. (**A**) qRT-PCR analysis of miR-21 expression in human breast cancer M231 control and PRL2 overexpression cells normalized to RNU-6 snRNA. Representative Western blots showing M231 cell lysates with Flag (PRL2) and β-Actin antibodies. Each sample was analyzed in triplicate, and the Student’s t-test was used to measure significance (***P < 0.001). (**B**) qRT-PCR analysis of miR-21 expression in human breast cancer MCF7 control and PRL2 overexpression cells, normalized to RNU-6 snRNA. Representative Western blots showing MCF7 cell lysates with Flag (PRL2) and β-Actin antibodies. Each sample was analyzed in triplicate, and the Student’s t-test was used to measure significance (***P < 0.001). (**C**) Relative proliferation rate of control and PRL2 overexpressing M231 cells in the absence or presence of miR-21 inhibitor. (**D**) Relative proliferation rate of control and PRL2 overexpressing MCF7 cells in the absence or presence of miR-21 inhibitor. (**E**) Relative wound healing rate of control and PRL2 overexpressing M231 cells in the absence or presence of miR-21 inhibitor. (**F**) Relative wound healing rate of control and PRL2 overexpressing MCF7 cells in the absence or presence of miR-21 inhibitor. Error bars indicate mean ± Standard Error of the Mean (SEM). Proliferation and wound healing assays were analyzed in three independent experiments, and the Student’s t-test was used to measure significance (*P < 0.05, **P < 0.01, ***P < 0.001).

Breast cancer is the second leading cause of cancer death among US women, and about 310,720 new cases of invasive breast cancer are expected to be diagnosed in 2024 (www.cancer.org). PRL2 mRNA is elevated in primary breast tumors relative to matched normal tissue and also dramatically higher in metastatic lymph nodes compared with primary tumors, suggesting that PRL2 plays a crucial role in breast cancer development [8]. Although it has previously been shown that there is a direct correlation between aberrant expression of miR-21 and breast cancer [48], it is unclear whether PRL2 can directly influence miR-21 expression in breast cancer. Considering that breast cancer is a highly heterogeneous disease, to evaluate the role of PRL2 on miR-21 in breast cancer cell lines with different molecular subtypes, we chose triple-negative breast cancer model MDA-MB-231 (M231) and ER-positive breast cancer model MCF7 cell lines and established M231 and MCF7 cells to stably express PRL2. We first confirmed that PRL2 overexpression is more than five-fold higher compared with endogenous PRL2 in M231 (5.9 fold) and MCF7 cells (5.5 fold) (Supplementary Figure S2A,B). We found that the levels of miR-21 were significantly higher in two independent PRL2 overexpressing cell lines compared with the control cells (Figure 2A,B). In addition to PRL2, overexpression of PRL1 and PRL3 can also up-regulate miR-21 expression in these cells (Supplementary Figure S2C,D). Moreover, Cmpd-43, a pan PRL inhibitor that disrupts PRL trimerization [49], markedly reduced miR-21 expression in M231 and MCF7 cells compared with the DMSO control, suggesting that PRL functionality is required for their effect on miR-21 expression (Supplementary Figure S2E,F). These data confirmed that PRL2 overexpression can up-regulate miR-21 in breast cancer cells.

We next evaluated the functional impact of PRL2 expression on breast cancer growth. Overexpression of PRL2 markedly promoted the proliferation of M231 and MCF7 cells (Figure 2C,D). Moreover, wound healing assays were conducted to explore the effect of PRL2 on breast cancer cell migration, a key determinant of malignant progression and metastasis. We found that the overexpression of PRL2 increased the migratory activity of M231 and MCF7 cells (Figure 2E,F). Next, we investigated whether the increased cell proliferation and migration induced by PRL2 expression are miR-21 dependent. We observed that miR-21 inhibition with a miR-21 inhibitor [50] can diminish both PRL2-mediated proliferation and migration in M231 and MCF7 cells (Figure 2C-2F). These results support a functional role for PRL2 in promoting breast cancer cell proliferation and migration via up-regulating miR-21 expression.

### PRL2 promotes miR-21 transcription through activation of STAT3

**Figure 3:**
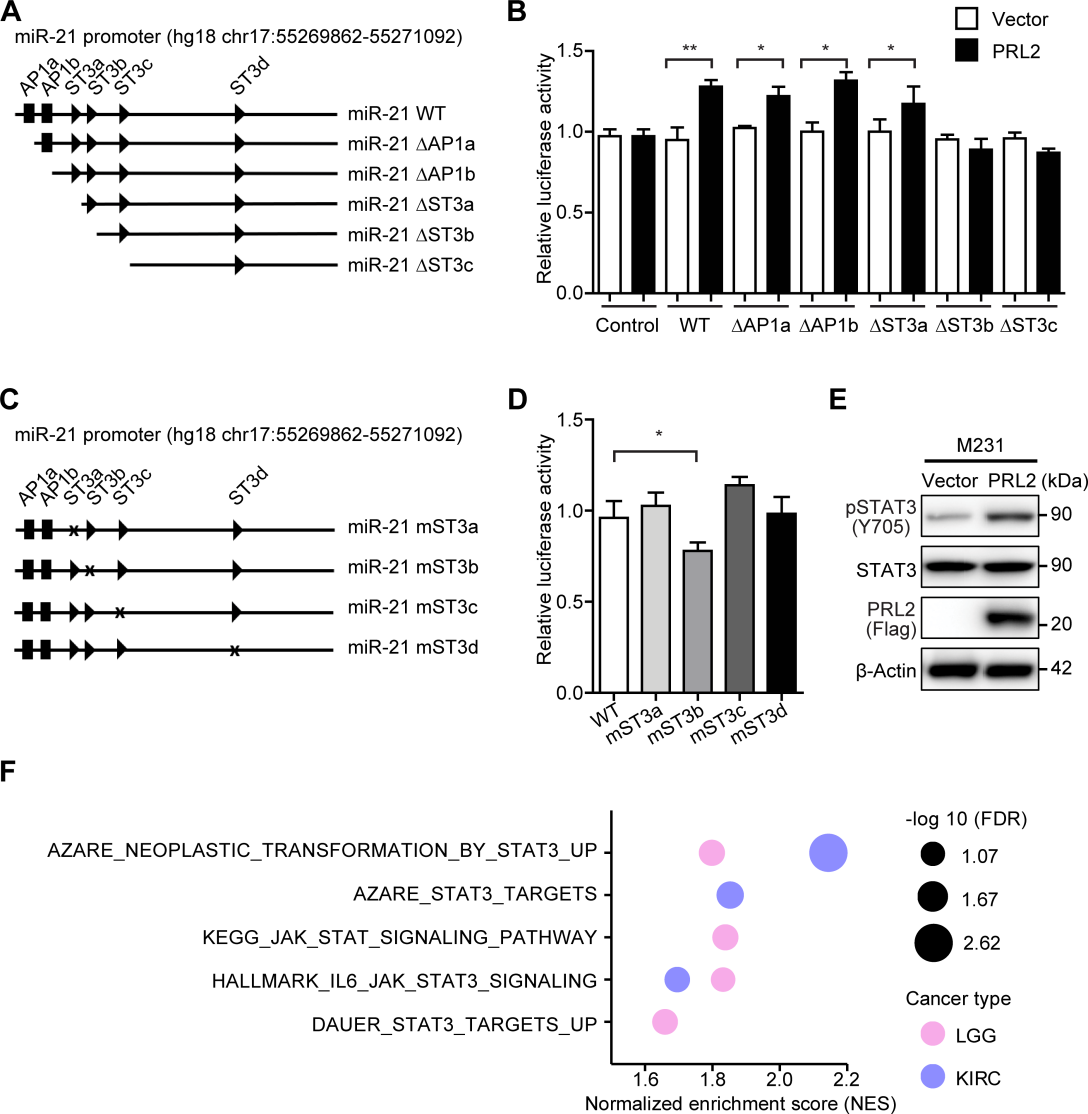
PRL2-mediated miR-21 up-regulation is transcriptionally regulated by STAT3. (**A**) Representation of the miR-21 promoter region for luciferase reporter assay. Constructs containing truncations of STAT3 and AP1 binding sites within the miR-21 promoter region are illustrated. (**B**) Quantification of luciferase assay for the miR-21 promoter region with constructs containing STAT3 and AP1 binding sites truncated mutants. Each sample was analyzed in three independent experiments, and the Student’s t-test was used to measure significance (*P < 0.05, **P < 0.01). (**C**) Mutagenesis of the miR-21 promoter region. Constructs featuring mutations in the four STAT3 binding sites within the miR-21 promoter region are shown. (**D**) Quantification of luciferase assay for the miR-21 promoter region with constructs containing STAT3 binding site mutants. Each sample was analyzed in three independent experiments, and the Student’s t-test was used to measure significance (*P < 0.05). (**E**) Western blotting showing STAT3 activation in control and PRL2 overexpressing M231 cells, detected by pSTAT3 (Tyr705), STAT3, PRL2, and β-Actin antibodies. (**F**) Gene Set Enrichment Analysis (GSEA) analysis of the Hallmark IL6–JAK–STAT3 pathway and other STAT3-related gene signature enrichment in both KIRC and LGG patients with high PRL2 expression.

Activating protein-1 (AP-1) and STAT3 are transcription factors that are capable of regulating miR-21 expression, and their activities are associated with cellular oncogenic properties [51–53]. To characterize the potential transcriptional regulation of the PRL2-mediated up-regulation of miR-21 by AP-1 and STAT3, we analyzed the miR-21 promoter activity using a dual-luciferase reporter assay [54]. The miR-21 promoter contains several putative AP-1 and STAT3 recognition sites (Figure 3A). Reporter plasmids containing either the WT miR-21 promoter or a series of mutant constructs containing truncations of AP-1 and STAT3 binding sites were transiently co-transfected with PRL2 in HEK293 cells (Figure 3B). We found that the reporter plasmids ΔAP1a (truncation that deletes the first AP1 binding site), ΔAP1b (truncation that deletes both AP1 binding sites), and ΔST3a (truncation that removes both AP1 binding sites and the first STAT3 binding site) had no effect on the PRL2-mediated luciferase reporter activity. In contrast, the luciferase activity was diminished upon transfection of the reporter plasmids ΔST3b (truncation that removes both AP1 binding sites and the first two STAT3 binding sites) or ΔST3c (truncation that removes both AP1 binding sites and the first three STAT3 binding sites) (Figure 3B), implicating ST3b as the functional site for STAT3 binding to the miR-21 promoter. Indeed, the luciferase activity was decreased only when the second STAT3 binding site was mutated (mST3b) (Figure 3C,D). These data indicate that the PRL2-mediated miR-21 transcription is regulated by the STAT3, but not AP1, binding site in the miR-21 promoter region, and that the ST3b site is the major contributor to its transcriptional activity.

STAT3 can be phosphorylated at Tyr705 by JAK2, leading to its dimerization and activation [40]. To further define the mechanism by which PRL2 stimulates the STAT3-mediated miR-21 expression, we examined whether PRL2 can promote STAT3 activation. As expected, we found that PRL2 markedly increased the levels of phospho-STAT3 (Tyr705) in M231 cells, confirming the ability of PRL2 to promote STAT3 activation (Figure 3E). To establish the clinical relevance of this PRL2-mediated STAT3 activation, we surveyed the TCGA cancer patient database. Gene Set Enrichment Analysis (GSEA) revealed that the Hallmark IL6–JAK–STAT3 pathway gene signature is enriched in both KIRC and LGG patients with high PRL2 expression (Figure 3F, Supplementary Figure S3A,B). In addition, the GSEA plots also showed that PRL2 expression positively correlated with other STAT3 gene signatures (KEGG JAK STAT SIGNALING PATHWAY; DAUER STAT3 TARGETS UP; AZARE NEOPLASTIC TRANSFORMATION BY STAT3 UP; AZARE STAT3 TARGETS) in these patients (Figure 3F, Supplementary Figure S3A,B), suggesting that PRL2 contributes to the activation of JAK2/STAT3 signaling in KIRC and LGG. Moreover, correlation analysis of the Clinical Proteomic Tumor Analysis Consortium (CPTAC) breast cancer proteome further supported the correlation between the PRL2 protein and STAT3 phosphorylation at Tyr705 in TCGA breast cancer patient samples (Supplementary Figure S3C) [55]. Taken together, our data suggest that PRL2 increases miR-21 transcription by promoting STAT3 activation.

### PRL2 promotes STAT3 activation by dephosphorylating JAK2 at Tyr570

**Figure 4:**
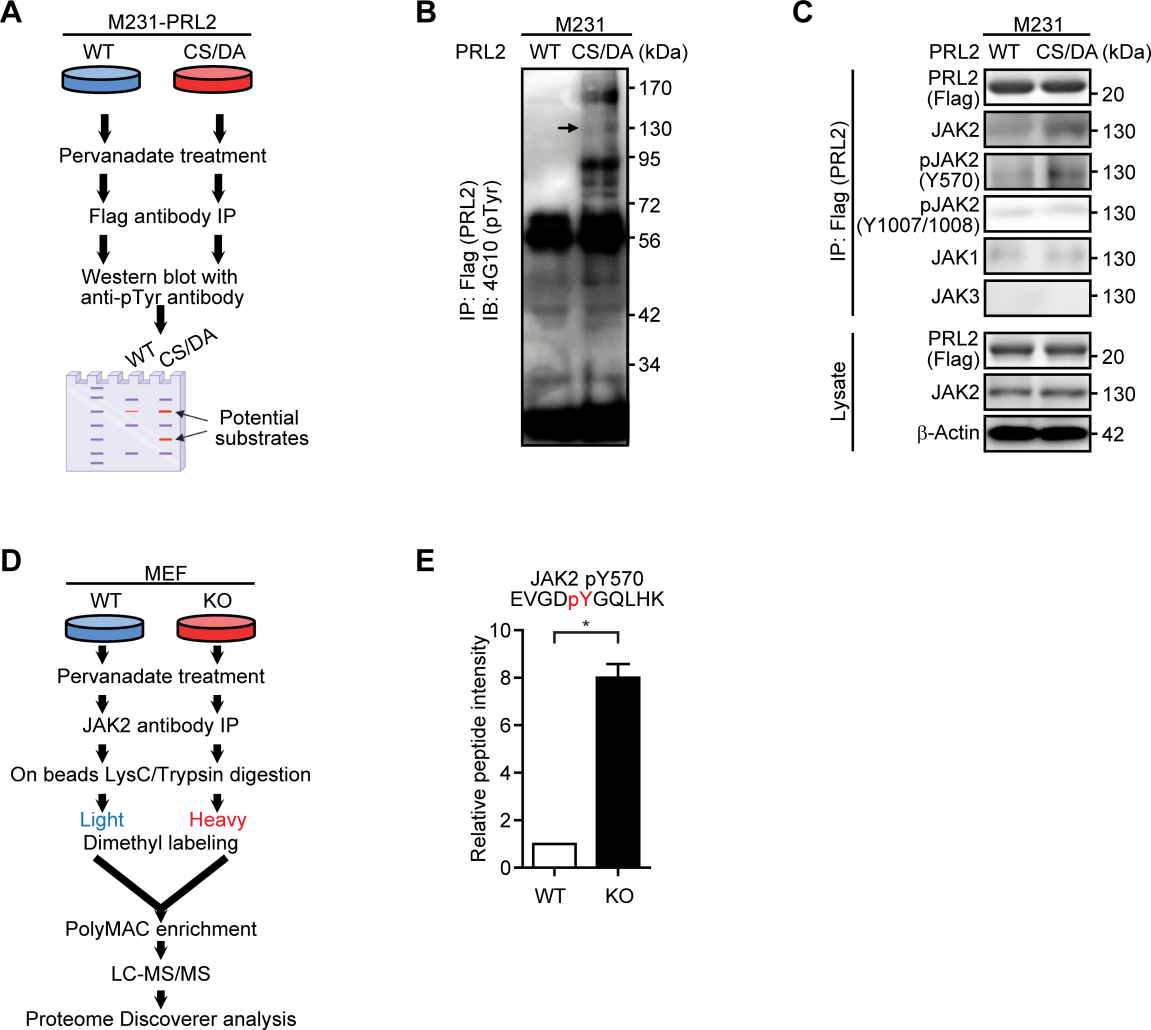
PRL2 dephosphorylates JAK2 at tyrosine 570 to promote STAT3 activation. (**A**) The experimental design of the substrate-trapping assay in M231 cells. M231 cells stably expressing PRL2 WT and CS/DA mutant were treated with pervanadate for 30 min and then replaced with fresh medium for another 30 min. The cells were lysed and immunoprecipitated by Flag beads and detected by anti-phospho-tyrosine antibody (4G10). (**B**) Western blotting showing the substrate trapping with PRL2 CS/DA mutant in M231 cells. The arrow indicates the potential JAK2 position. (**C**) Substrate-trapping samples from M231 cells stably expressing PRL2 WT or PRL2 CS/DA were analyzed by Western blot using Flag (PRL2), JAK2, pJAK2(Y570), pJAK2(Y1007/Y1008), JAK1, JAK3 and β-Actin antibodies. (**D**) The experimental design of JAK2 immunoprecipitation followed by the LC-MS/MS analysis in MEF cells. PRL2 WT and KO MEF cells were treated with pervanadate for 30 min and then incubated with fresh medium for another 30 min. The cells were lysed and immunoprecipitated by JAK2 antibody and analyzed by LC-MS/MS. (**E**) Quantification of JAK2 Tyr570 containing peptide in PRL2 WT and KO MEF cells identified by the LC-MS/MS. Student’s t-test was used to measure significance (*P < 0.05).

Given that the phosphorylation of Tyr570 within the inhibitory JH2 domain inhibits JAK2-mediated STAT3 activation [41,42], we investigated whether PRL2 could directly dephosphorylate phosphoJAK2 at Tyr570 to activate its kinase activity for STAT3 activation. We performed substrate-trapping experiments to examine whether JAK2 is a potential substrate of PRL2. In these experiments, M231 cells overexpressing WT PRL2 or the catalytically inactive PRL2 mutant (C101S/D69A) were first treated with pervanadate and then Flag-PRL2 or its mutant were immunoprecipitated and immunoblotted with anti-phosphotyrosine antibodies (Figure 4A). Among the tyrosine-phosphorylated proteins captured by the PRL2 substrate-trapping mutant was a 130 kDa protein, which corresponds to the molecular weight of JAK2 (Figure 4B). To ascertain whether JAK2 is indeed a substrate of PRL2, a JAK2-specific antibody was used to directly detect JAK2 in the substrate-trapping samples. As expected, the PRL2 CS/DA mutant samples showed higher levels of JAK2 and pJAK2 (Tyr570) (Figure 4C). In contrast, no change in JAK1, JAK3, and pJAK2 (Tyr1007/Tyr1008) was detected in the samples (Figure 4C).

To further confirm the PRL2-mediated dephosphorylation of JAK2, we performed liquid chromatography-mass spectrometry/mass spectrometry (LC-MS/MS) experiments by immunoprecipitating JAK2 from either PRL2 WT or PRL2 KO MEF cells with JAK2 antibody (Figure 4D). The immunoprecipitated JAK2 samples were trypsin digested on-beads, and stable-isotope dimethyl labeling was performed on JAK2 peptides from both the WT MEF cell group (light, ^12^CH_2_O) and PRL2 KO MEF cell group (heavy, ^13^CD_2_O). The overall phosphorylation was determined by immobilized metal affinity chromatography (IMAC) enrichment followed by the LC-MS/MS analysis. In agreement with our biochemical results, JAK2 Tyr570 was the only phosphorylation site identified and an ~eight-fold increase in JAK2 Tyr570 phosphorylation was observed upon PRL2 deletion in MEFs (Figure 4E, Supplementary Figure S4). Taken together, these data suggest that PRL2 can promote JAK2-mediated STAT3 activation by specific dephosphorylation of JAK2 at Tyr570, leading to increased miR-21 expression.

### PRL2 reduces PTEN expression in a miR-21-dependent post-transcriptional mechanism

**Figure 5:**
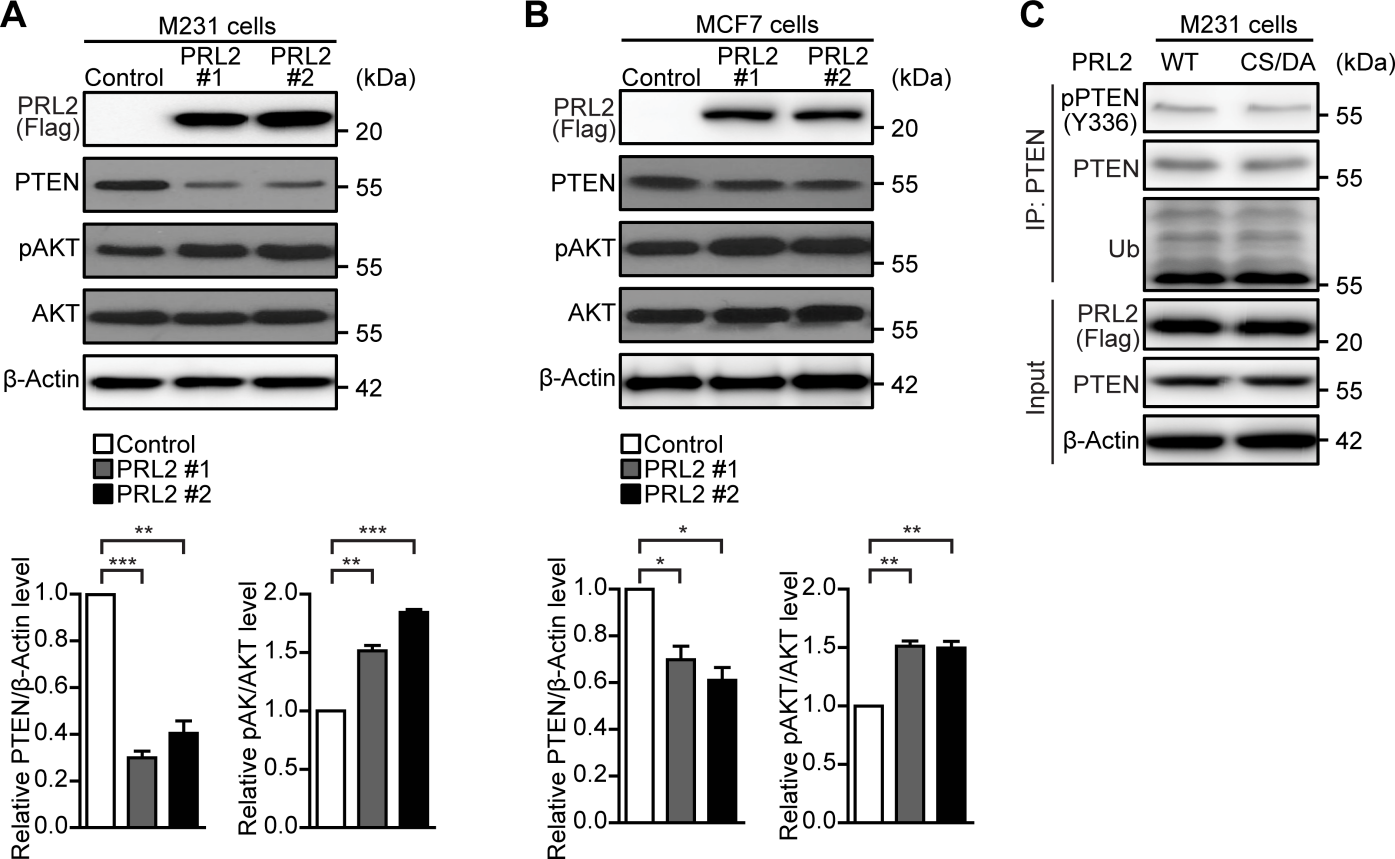
PRL2 can down-regulate PTEN in both miR-21- and NEDD4-dependent mechanisms. (**A)** PRL2 down-regulates PTEN and promotes AKT phosphorylation in M231 cells. Western blotting showing PTEN, pAKT, and AKT expression in control and PRL2 overexpressing M231 cells. Quantifications were performed on the relative PTEN/β-Actin level and the relative pAKT/AKT level from control and PRL2 overexpressing M231 cells in three independent experiments, and the Student’s t-test was used to measure significance (**P < 0.01, ***P < 0.001). (**B**) PRL2 down-regulates PTEN and promotes AKT phosphorylation in MCF7 cells. Western blotting showing PTEN, pAKT, and AKT expression in control and PRL2 overexpressing MCF7 cells. Quantifications were performed on the relative PTEN/β-Actin level and the relative pAKT/AKT level from control and PRL2 overexpressing MCF7 cells in three independent experiments, and the Student’s t-test was used to measure significance (*P < 0.05, **P < 0.01). (**C**) Western blot showing PTEN phosphorylation and ubiquitination in PTEN immunoprecipitated samples from M231 cells stably expressing either WT PRL2 and or CS/DA mutant. PTEN (Tyr336), PTEN, Ubiquitin, Flag (for PRL2), and β-Actin antibodies were used.

The level of the PTEN tumor suppressor can be regulated by various molecular mechanisms, including transcriptional regulation, post-translational modifications, and post-transcriptional regulation [56]. Since miR-21 functions to curtail the expression of tumor suppressor genes, including PTEN [35], we speculated that PRL2 might also be able to reduce PTEN expression through a post-transcriptional mechanism by up-regulation of miR-21. Consistent with our findings that PRL2 increases miR-21 expression in breast cancer cells (Figure 2A,B), overexpression of PRL2 markedly down-regulated PTEN and increased AKT phosphorylation in both M231 and MCF7 cells (Figure 5A,B). We previously found that PRL2 could also down-regulate PTEN through directly dephosphorylating PTEN at Tyr336, leading to enhanced NEDD4-mediated PTEN polyubiquitination and degradation [19]. Interestingly, no change in PTEN phosphorylation and polyubiquitination was observed in M231 cells harboring either WT PRL2 or its CS/DA catalytically inactive mutant (Figure 5C). This finding suggests that the PRL2-mediated NEDD4-dependent post-translational regulation of PTEN may occur in a cell-type-specific manner and that the post-transcriptional regulation of PTEN by the PRL2-mediated up-regulation of miR-21 was likely the dominant mechanism in M231 cells.

**Figure 6:**
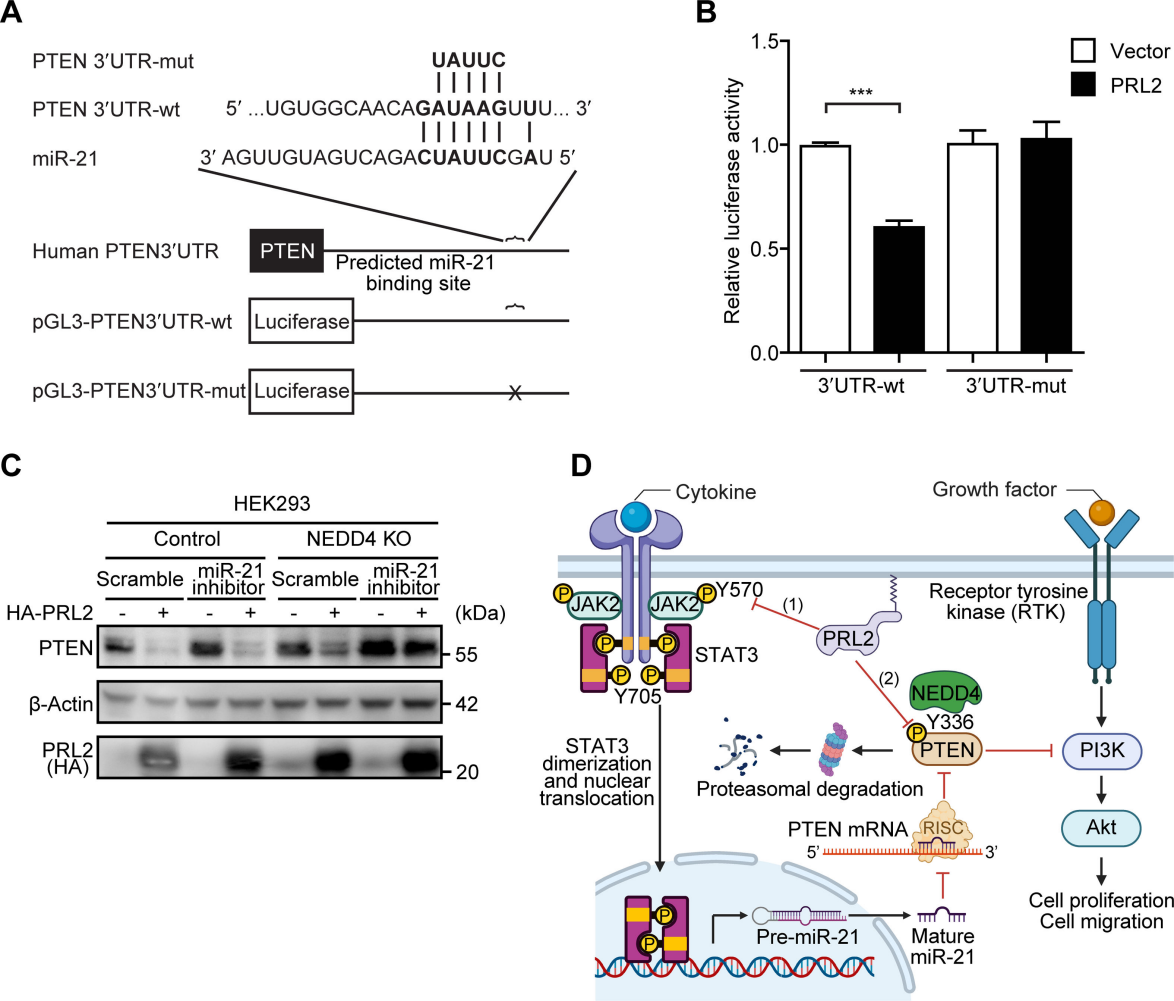
PRL2 down-regulates PTEN through the up-regulation of miR-21 binding to the 3′-UTR of PTEN. (**A**) Representation of the pGL3-PTEN 3′-UTR reporter plasmid construction. Sequences shown are computationally predicted binding sites for miR-21 in the PTEN 3′-UTR. (**B**) Quantification of luciferase assay of the PTEN 3′-UTR in vector control and PRL2 overexpressing HEK293 cells in three independent experiments and the Student’s t-test was used to measure significance (***P < 0.001). (**C**) Western blot showing PRL2 down-regulates PTEN in the absence or presence of miR-21 inhibitor in control and NEDD4 KO HEK293 cells, and detected by PTEN, β-Actin, and HA (PRL2) antibodies. (**D**) Proposed model on the dual functions of PRL2 in the PTEN down-regulation. (1) JAK2/STAT3-miR-21-PTEN pathway. Upon cytokine, growth factors, and hormones binding to its receptor, JAK2 is activated, resulting in STAT3 phosphorylation, dimerization, and nuclear translocation, where it acts as a transcription factor to promote the expression of miR-21. Once outside of the nucleus, miR-21 will mature and join a RISC complex and then target the 3′-UTR of PTEN mRNA, resulting in decreased expression of PTEN protein product and increased AKT activation, eventually leading to increased cell proliferation and migration. PRL2 directly dephosphorylates JAK2 at Tyr570, a negative regulatory tyrosine residue responsible for JAK2 activation, allowing the full activation of JAK2/STAT3 signaling and enhanced miR-21 expression, leading to PTEN down-regulation and cancer progression. (2) NEDD4-PTEN pathway. PRL2 directly dephosphorylates PTEN at Tyr336 and promotes the interaction between PTEN and its E3 ligase NEDD4, leading to increased poly-ubiquitination and proteasome degradation.

To further confirm that PTEN can be regulated by PRL2 through miR-21 induction, we examined whether miR-21 directly binds to the 3′-UTR of PTEN mRNAs in the presence of PRL2 by the luciferase reporter assay (Figure 6A). The 3′-UTR of PTEN mRNA containing the potential binding sites for miR-21, together with their corresponding mutated sequences, was co-transfected with PRL2 into HEK293 cells. A marked decrease in the relative luciferase activity was noticed in WT PTEN 3′-UTR, but not in mutant PTEN 3′-UTR constructs, when PRL2 was overexpressed in HEK293 cells, indicating that overexpression of PRL2 promotes miR-21 levels, which directly binds to the PTEN 3′-UTR to inhibit PTEN expression (Figure 6B). To determine if the PRL2-mediated PTEN down-regulation can also be triggered by PRL2-induced miR-21 up-regulation, we examined the PTEN level upon PRL2 overexpression in NEDD4 deleted HEK293 cells (Figure 6C), where the NEDD4-mediated PTEN degradation mechanism is abolished [19]. As expected, overexpression of PRL2 is still able to down-regulate PTEN even in the absence of NEDD4, suggesting a NEDD4-independent mechanism by which PRL2 can control PTEN expression (Figure 6C). This PRL2-mediated PTEN down-regulation relies on miR-21 since the miR-21 inhibitor could abolish PRL2 overexpression-mediated PTEN alteration (Figure 6C). Collectively, our findings suggest that PRL2 can down-regulate PTEN through up-regulation of miR-21 through dephosphorylation of JAK2 at Tyr570 and activation of STAT3 (Figure 6D).

## Discussion

Although overexpression of the PRLs is associated with the progression of multiple cancers [3,8,44], the specific mechanisms by which these phosphatases promote tumorigenesis have so far remained unclear. We and others have previously reported that PRL expression can activate a number of signaling pathways in cell culture, including the Rho family of small GTPases, Src, STAT3, ERK1/2, and PI3K/AKT [6,43,47]. We have found that the deletion of PRL2 in mice causes placental insufficiency, impaired spermatogenesis, and defects in hematopoietic stem cell self-renewal due to an increased PTEN protein level and decreased AKT activation [11,13–15]. These observations suggest that PRL2 is a negative regulator of PTEN. As one of the most inactivated tumor suppressors, PTEN is highly regulated by genetic mutations, epigenetic silencing, transcriptional repression, microRNA processing, and post-translational mechanisms [56]. Unlike classical tumor suppressors, partial loss of PTEN is regularly observed in a considerable number of human cancers [20–22]. Consequently, the ability of PRL2 to reduce PTEN expression provides a biochemical basis for its oncogenic propensity.

We previously showed that PRL2 can post-translationally down-regulate PTEN by directly dephosphorylating PTEN at Tyr336, thereby increasing its association with NEDD4 for polyubiquitination and degradation [19]. Given the various ways that PTEN can be regulated [25,26,56], we considered whether the PRLs can also control PTEN expression through additional means. miRNAs are key post-transcriptional regulators of gene expression that work by silencing target transcripts through base-pair complementation [30]. Accordingly, the dysregulation of miRNAs has been associated with many diseases, including cancers [34]. In the present study, we discovered a NEDD4-independent mechanism by which PRL2 reduces PTEN expression via up-regulation of miR-21 (Figure 6D). As a crucial regulator of several proliferative pathways, miR-21 is transcriptionally regulated by the JAK2/STAT3 pathway and elevated in all human cancers [34,36–39]. Notably, miR-21 functions to curtail the expression of many target genes, including PTEN [35]. In addition, inhibition of miRNA-21 has been shown to increase PTEN levels and subsequently reduce cell migration and invasion in multiple types of cancer [57–59].

Here, we established a linkage between PRL2 expression and those of both miR-21 and PTEN. We found that PRL2-mediated miR-21 up-regulation contributes to its oncogenic properties. We determined that the underlying mechanism by which PRL2 up-regulates miR-21 is through the dephosphorylation of JAK2 at Tyr570, leading to STAT3 activation. We then demonstrated that the PRL2-mediated STAT3 activation is responsible for reduced PTEN expression through a miR-21-dependent post-transcriptional mechanism. Our findings not only link PRL2 to miR-21 regulation but also provide additional evidence that PRL2 serves as a negative regulator of PTEN. Given that even a slight reduction in PTEN levels can significantly increase tumor formation [20,60], the ability of PRL2 to down-regulate PTEN, through both a miR-21-dependent post-transcriptional mechanism and the previously reported NEDD4-dependent post-translational PTEN regulation [19], contributes to its oncogenic potential to enhance cancer progression. As shown in our recent studies [19,23,24], we believe that therapeutic targeting of PRL2 represents a promising strategy to augment PTEN function for cancer treatment.

## Materials and methods

### Cell culture and transfection

Human HEK293 (ATCC, CRL-1573), MCF7 (ATCC, HTB-22), M231 (ATCC, HTB-26), and mouse GC-1 (ATCC, CRL-2053) cell lines were purchased from ATCC. The cells were grown in Dulbecco’s modified Eagle’s medium (DMEM) (Gibco, CA, USA) supplied with 10% fetal bovine serum (FBS) and 100 U/ml penicillin and 100 μg/ml streptomycin in a humidified atmosphere of 5% CO_2_ at 37°C. PRL2 was silenced in HEK293 cells through CRISPR/Cas9 editing using pSpCas9(BB)-2A-Puro (PX459) V2.0 (a gift from Feng Zhang, Addgene plasmid #62988) with sgRNA sequences designed via a gRNA design tool (Feng Lab CRISPR Design Web Tool at http://crispr.mit.edu). miR-21 inhibitor and respective negative control oligonucleotides were purchased from Qiagen. Transfection was performed using Lipofectamine 3000 (Invitrogen) according to the manufacturer’s recommendations.

### TCGA analysis

TCGA level 3 RNAseqV2 gene expression, reverse phase protein array (RPPA), and clinical data of primary patient clinical samples from KIRC (*n* = 587), brain LGGs (*n* = 526), and testicular germ cell tumors (TGCT, *n* = 156) were downloaded from the Broad Institute’s Firehose (http://gdac.broadinstitute.org). Patients were separated into two subgroups (low PRL2 and high PRL2) based on their PRL2 mRNA expression levels using the mean value for the miR-21 level analysis and the survival analysis with the Mann-Whitney tests and the Kaplan–Meier survival analysis. The correlation coefficient between PRLs mRNA and miR-21 level was measured by the Spearman rank correlation analysis. GSEA was performed by the GSEA v4.2.2 software (http://www.gsea-msigdb.org/gsea/index.jsp).

### Immunoprecipitation and immunoblotting

Tissues or cultured cells were harvested and lysed with ice-cold lysis buffer [50 mM Tris (pH 8.0, 150 mM NaCl, 10% Glycerol, 1% Triton-X-100], supplemented with a complete protease inhibitor tablet and PhosSTOP tablets (Roche Applied Science, Indianapolis, IN, USA). The protein concentrations were measured using a BCA protein assay kit (Thermo Fisher Scientific, Rockford, IL, USA) according to the manufacturer’s instructions. For immunoprecipitation, antibody and protein A/G beads were then added to cell lysates and incubated at 4°C for 3 h to overnight followed by extensively washing with the lysis buffer. Proteins on the resins were eluted with SDS sample buffer and then subjected to analysis by SDS-PAGE followed by immunoblotting (Western blot) with appropriate antibodies. For immunoblotting, equal amounts of protein were separated by SDS-PAGE gel and transferred to a nitrocellulose membrane. The membranes were incubated with the primary antibodies and then with horseradish peroxidase (HRP)-conjugated secondary antibodies (Cell Signaling, Denver, MA, USA). Finally, the immunoreactive protein bands were detected using an enhanced chemiluminescence (ECL) kit (Pierce, Rockford, IL, USA). The following antibodies were used: AKT (#2920, 1:1000), pAKT (Ser473, #4060, 1:1000), pAKT (Thr308, #13038, 1:1000), PTEN (#9188, 1:1000), STAT3 (#9139, 1:1000), pSTAT3 (Tyr705, #9145, 1:1000), Ubiquitin (#3936, 1:1000), JAK1 (#3344, 1:1000), JAK3 (#8827, 1:1000) from Cell Signaling; pPTEN (Tyr336, F48744, 1:500) from NSJ Bioreagents; pJAK2 (Tyr570, PA5-104705, 1:500) from Thermo Fisher Scientific; phosphor tyrosine (4G10, 05–321, 1:2000) from Millipore Sigma; β-Actin (sc-47778, 1:200), JAK2 (sc-390539, 1:200), and HA (sc-7392, 1:200) from Santa Cruz; and Flag M2 affinity gel (F1804) from Sigma Aldrich. The bands were scanned in the linear range, and the intensity was quantified by ImageJ.

### RNA isolation and quantitative Real-Time PCR (qRT-PCR)

RNA was isolated using an RNA isolation kit (Qiagen), and the first-strand cDNA synthesis was performed with the Superscript III reagent (Invitrogen). Quantitative real-time PCR was performed in triplicate using the SYBR Green I qPCR master mix on a LightCycler 96 (Roche). Expression analysis in different cell types was performed using specific primers for each gene (Supplementary Table S1). The PCR products were assessed by melting curve analysis, and gene expression levels were calculated using the ^ΔΔ^C_t_ method after normalization to the RNU-6 or GAPDH housekeeping gene, which remained the same in different treatment groups.

### Luciferase reporter assay

The full-length WT and mutated (Mut) PTEN 3′-UTR containing putative binding sites for miR-21 were obtained by PCR using the following primers: WT (forward: 5′CCGGCTCGAGATCTCCCACATTCATACC3′; reverse: 5′TAAGCGGCCGCTTCATTAGCAGAAACCC3′) and mutated type (forward: 5′GTCAAAGGGGCCTGAGAAAAGAATG3′; reverse: 5′TAAGTTGCACCTCAGAGTGCAAACA3′). The WT and mutated PTEN 3′-UTR were inserted into the pmir-GLO dual-luciferase miRNA target expression vector (Promega, Madison, WI, USA). All vectors were verified by DNA sequencing. Cells were seeded into 24-well plates and incubated for 24 h. The cells were then co-transfected with a luciferase reporter vector with PRL2 vector or negative control vector. Forty-eight hours after transfection, luciferase activity was measured using the Dual-Luciferase Reporter Assay System (Promega) according to the manufacturer’s instructions.

### MTT proliferation assay

A total of 5 × 10^3^ cells were seeded into 96-well culture plates. After transfection, the cells were incubated with 5 mg/ml MTT (Sigma) at 37°C for 4 h. Then, the medium was removed, and dimethyl sulfoxide (DMSO) was added to each well. The optical density (OD) was determined with spectrophotometry at a wavelength of 540 nm.

### Wound healing assay

The uniform scratches were created using silicon culture inserts with three individual wells for cell seeding according to the manufacturer’s instructions. After removing the inserts, the cell was washed to remove the floating cells, and a fresh medium containing 5% FBS was added. The photos were captured by a Nikon microscope at 0 and 24 h under a 10× magnification. Wound healing ability was quantified by measuring the relative wound closure compared with vector control cells at 24 h.

### Protein extraction and digestion

Beads with proteins immunoprecipitated were lysed in 12 mM SDC-SLS: 100 mM Tris-HCl (pH 8.5), 12 mM sodium deoxycholate (SDC), and 12 mM sodium lauroyl sarcosinate (SLS). Proteins were reduced and alkylated with 10 mM tris-(2-carboxyethyl)phosphine (TECP) and 40 mM chloroacetamide (CAA) at 95°C for 5 min and then centrifuged down for 2–3 min. The supernatant containing reduced and alkylated proteins was transferred into a new Eppendorf tube. The supernatant was diluted five-fold with 50 mM triethylammonium hydrogen carbonate buffer (TEAB) and digested with 1 µl 2.5 AU/ml Lys-C (Wako) for 3 h at 37°C and 500 ng trypsin (Sigma) overnight at 37°C. The SDC and SLS were removed by adding 100% acetyl acetate in a 1:1 (vol/vol) sample to acetyl acetate volume ratio. Samples were acidified with 10% trifluoroacetic acid (TFA) to a pH of ~3 and desalted by a homemade stage tip with the styrene divinyl benzene (SDB-XC) membrane (3M).

### Dimethyl labeling

Every 50–100 µg of tryptic peptides were dissolved in 100 µl of 100 mM TEAB and were mixed with 4 µl of 4% ^13^CD_2_O (PRL2 KO MEFs) or ^12^CH_2_O (PRL2 WT MEFs), and then 4 µl of freshly prepared 0.6 M sodium cyanoborohydride was immediately added [61]. The mixture was agitated for 1 h at room temperature. The reaction was quenched by adding ice-cold 16 µl of 1% ammonium hydroxide and agitating the mixture for 1 min. Heavy and light dimethyl-labeled peptides were mixed after the labeled peptides were acidified with 20 µl of 10% formic acid and then desalted using a homemade stage tip with the styrene divinyl benzene (SDB-XC) membrane (3M).

### PolyMAC-Ti enrichment of phosphopeptides

PolyMAC-Ti kit contains PolyMAC-Ti bead slurry, loading, washing, and elution buffer, and tip with a frit (Tymora Analytical, IN) [62]. Peptides were dissolved in 200 μl of loading buffer and incubated with 50 μl of the PolyMAC-Ti bead slurry for 20 min with gentle agitation. The bead slurries were loaded with samples into a frit-containing tip. The beads in the tip were washed twice with 200 μl of loading buffer and then once with 200 μl of wash buffer. Each wash involved centrifugation at 20 × *g* for 2 min at room temperature, followed by 100 × *g* for 1 min or until the beads were completely dried before adding the next buffer. The phosphopeptides were then eluted from the beads using two incubations with 50 μl of elution buffer using the same centrifuge settings. The eluates were collected and dried in a vacuum centrifuge.

### LC-MS/MS analysis

Enriched phosphopeptides were dissolved in 5 µl of 0.3% formic acid (FA) with 3% ACN. Four microliters of the enriched phosphopeptides were injected into an Easy-nLC 1000 liquid chromatography system (Thermo Fisher Scientific). Peptides were separated on a 45 cm in-house packed column (360 µm OD × 75 µm ID) containing C18 resin (2.2 µm, 100 Å, Michrom Bioresources) with a 30 cm column heater (Analytical Sales and Services) set at 60°C. The mobile phase buffer consisted of 0.1% FA in ultrapure water (buffer A). Samples were eluted using a 60 min gradient of 5%–30% buffer B (0.1% FA in 80% ACN) at a flow rate of 250 nl/min. The Easy-nLC 1000 was coupled online with a Velos Pro LTQ-Orbitrap mass spectrometer (Thermo Fisher Scientific). The mass spectrometer was operated in the data-dependent mode in which a full MS scan (from m/z 350–1500 with the resolution of 30,000 at m/z 400) was followed by MS/MS of the 15 most intense ions being subjected to collision-induced dissociation (CID) fragmentation (normalized collision energy – 35%, automatic gain control – 3E4, and max. injection time – 100 ms).

### Database searching

The raw files were searched directly against the *Homo sapiens* database with no redundant entries (Uniprot FASTA file released August 2017) using the SEQUEST HT algorithm and Byonic search engine built into Proteome Discoverer 2.2 (Thermo Fisher Scientific) with a 1% FDR cutoff at protein and peptide. The first peptide precursor mass tolerance was set at 20 ppm, and MS/MS tolerance was set at 0.6 Da. Search criteria included a static carbamidomethylation of cysteines and variable modifications of oxidation on methionine residues, acetylation at the N-terminus of proteins, and phosphorylation on serine, threonine, and tyrosine residues for the identification of phosphorylation sites. The search was performed with full tryptic digestion and allowed a maximum of two missed cleavages on the peptides analyzed from the sequence database. Dimethyl-labeling quantitation was performed by setting the multiplicity as 2 (DimethLys0 and DimethNter0; DimethLys6 and DimethNter6).

### Quantification and statistical analysis

Western blots were quantified by densitometry using ImageJ (https://imagej.net/ij/) version 1.48 v. For cell-based MTT proliferation assays, wound healing assays, luciferase assays, Western blots, and qRT-PCR analysis, the Student’s *t*-test was used to measure the significance, as indicated in the figure legends. Statistical tests were performed with Prism (GraphPad Software) version 9.5.0. Unless specified otherwise, error bars in figures indicate mean ± standard deviation (SD) from at least three independent experiments. A *P*-value less than 0.05 was considered statistically significant.

## Supplementary material

online supplementary material 1.

## Data Availability

All data and reagents are available from the authors upon request.

## Abbreviations

AP-1: Activating protein-1
CPTAC: Clinical proteomic tumor analysis consortium
GSEA: Gene set enrichment analysis
JAK2: Janus kinase 2
KIRC: kidney renal clear cell carcinoma
LC MS/MS: Liquid chromatograp hy-mass spectrometry/mass spectrometry
LGG: Low-grade gliomas
M231: MDA-MB-231
MEF: Mouse embryonic fibroblast
PRLs: phosphatases of regenerating liver
PTP: protein tyrosine phosphatase
STAT3: Signal transducer and activator of transcription 3
TCGA: The cancer genome atlas
TGCT: Testicular germ cell tumors.
miR-21: MicroRNA-21
