# Supplementary material for: The PRL2 phosphatase up-regulates miR-21 through activation of the JAK2/STAT3 pathway to down-regulate the PTEN tumor suppressor
Source: Biochem J. 2025 Apr 11;482(7):BCJ20240626. doi: 10.1042/BCJ20240626 (PMC12198624; doi:10.1042/BCJ20240626)
Supplement: online supplementary material 1. [file bcj-482-07-bcj-2024-0626-s001.docx]

**The PRL2 Phosphatase Upregulates miR-21 through Activation of the JAK2/STAT3 Pathway to Downregulate the PTEN Tumor Suppressor**

Qinglin Li^a#+^, Yunpeng Bai^a#^, Sarah M. Cavender^a^, Yiming Miao^a^, Frederick Nguele Meke^a^, Emily L Lasse-Opsahl^a^, Peipei Zhu^b^, Gina M. Doody^f^, W. Andy Tao^b,c,d,e^, and Zhong-Yin Zhang^a,c,d,e^*

^a^Borch Department of Medicinal Chemistry and Molecular Pharmacology, ^b^Department of Biochemistry, ^c^The James Tarpo Jr. and Margaret Tarpo Department of Chemistry, ^d^Purdue Institute for Cancer Research, and ^e^Purdue Institute for Drug Discovery, Purdue University, 720 Clinic Drive, West Lafayette, IN 47907, USA, ^f^Division of Haematology and Immunology, Leeds Institute of Medical Research, University of Leeds, UK,

#These authors contributed equally to the study.

*To whom correspondence should be addressed: zhang-zy@purdue.edu.

+Current Address: Thermo Fisher Scientific, 5781 Van Allen Way, Carlsbad, CA 92008, USA

Key words: PRL2, miR-21, PTEN, JAK2, STAT3

Table S1. qPCR primers sequence list:

| PTEN 3UTR-mut-5F | TTGTGGCAACAGTATTCTTTGCAGTTG |
| --- | --- |
| PTEN 3UTR-mut-3r | CAACTGCAAAGAATACTGTTGCCACAA |
| Ap1a-mut-5fn | TTGGATAAGGAAAACCTACAGATTGTCCTAATA |
| Ap1a-mut-3Rn | TATTAGGACAATCTGTAGGTTTTCCTTATCCAA |
| Ap1b-mut-5f | ATAAGGACTTAGATAAACCTAGACCGCCCCCTCTGA |
| Ap1b-mut-3R | TCAGAGGGGGCGGTCTAGGTTTATCTAAGTCCTTAT |
| ST3a-mut-5f | TCAAACCAGTTGGGCCGGGCCCTAGTGGTGATAAAT |
| St3a-mut-3r | ATTTATCACCACTAGGGCCCGGCCCAACTGGTTTGA |
| St3b-mut-5 | ATAAATGTGGGACCCCGGAGGGGTCATTCATTTTATTCT |
| St3b-mut-3r | AGAATAAAATGAATGACCCCTCCGGGGTCCCACATTTAT |
| ST3c-mut-5 | TTTTTTTTTTTTTGGCAACCCCAAAGGGGTCACAGAATT |
| ST3c-mut-3r | AATTCTGTGACCCCTTTGGGGTTGCCAAAAAAAAAAAAA |
| St3d-mut-5f | AACTTCTGTACATCGGCTCCCAAAAACAAGGGTAGA |
| St3d-mut-3r | TCTACCCTTGTTTTTGGGAGCCGATGTACAGAAGTT |

**Figure S1. The PRLs promote miR-21 expression.**


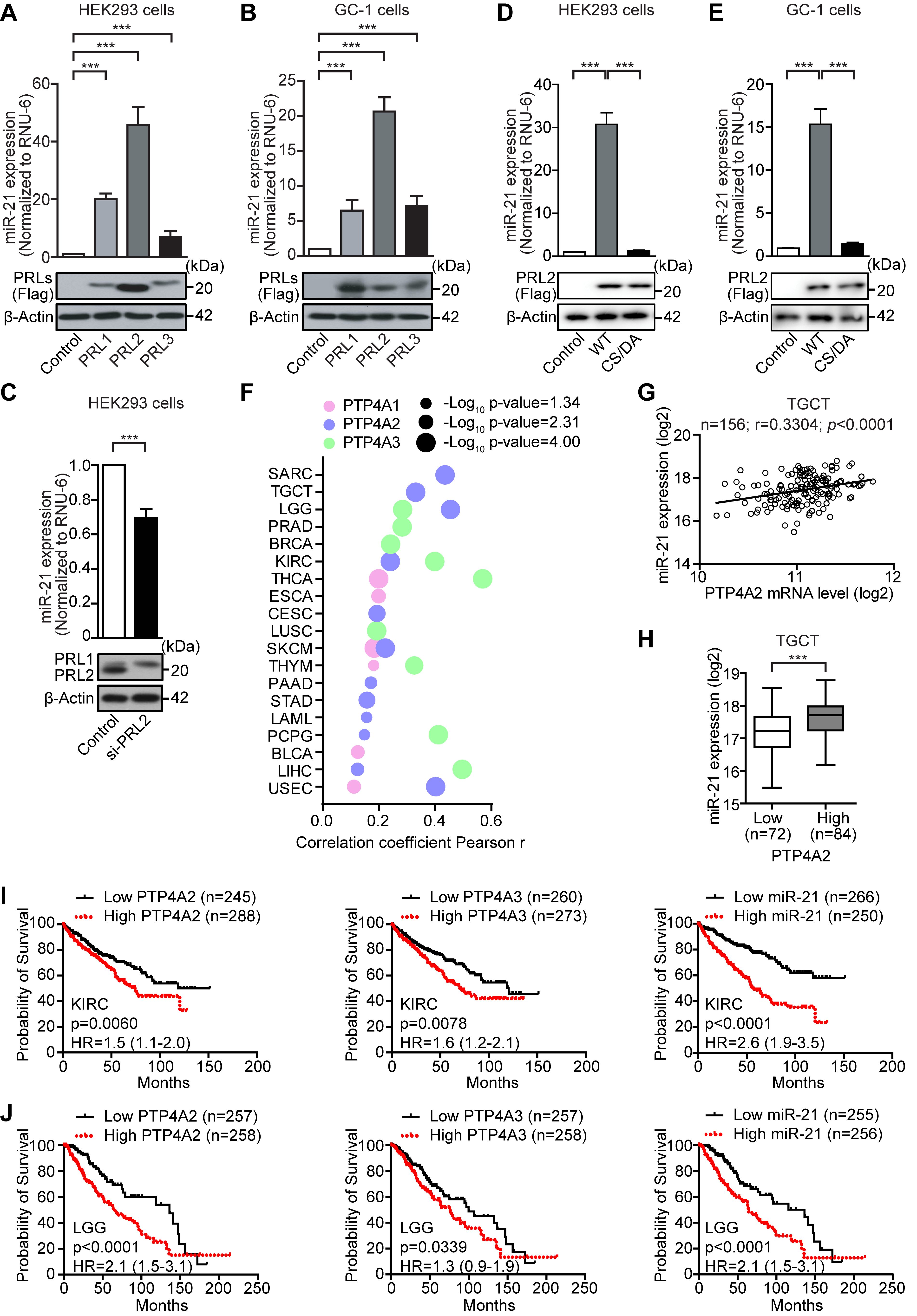


**A**. qRT-PCR analysis of miR-21 expression in HEK293 cells stably expressing control, PRL1, PRL2 and PRL3, normalized to RNU-6 snRNA. Each sample was analyzed in triplicate, and the Student t test was used to measure significance (***p<0.001). Western blotting showing PRLs protein expression in PRLs-Flag or control vector. **B**. qRT-PCR analysis of miR-21 expression in GC-1 cells stably expressing control, PRL1, PRL2 and PRL3, normalized to RNU-6 snRNA. Each sample was analyzed in triplicate, and the Student t test was used to measure significance (***p<0.001). Western blotting showing PRLs protein expression in PRLs-Flag or control vector. **C**. qRT-PCR analysis of miR-21 expression in HEK293 cells transiently expressing control and PRL2 siRNA, normalized to RNU-6 snRNA. Each sample was analyzed in triplicate, and the Student t test was used to measure significance (***p<0.001). Western blotting showing PRL2 protein expression in HEK293 cells. **D**. qRT-PCR analysis of miR-21 expression in HEK293 cells stably expressing control, PRL2 WT and CS/DA mutant, normalized to RNU-6 snRNA. Each sample was analyzed in triplicate, and the Student t test was used to measure significance (***p<0.001). Western blotting showing PRL2 and PRL2 (CS/DA) mutant protein expression in HEK293 cells. **E**. qRT-PCR analysis of miR-21 expression in GC-1 cells stably expressing control, PRL2 WT and CS/DA mutant, normalizing to RNU-6 snRNA. Each sample was analyzed in triplicate, and the Student t test was used to measure significance (***p<0.001). Western blotting showing PRL2 and PRL2 (CS/DA) mutant protein expression in GC-1 cells. **F**. The positive Pearson rank correlation between mRNA level of PTP4A1, PTP4A2 or PTP4A3 and miR-21 from a panel of TCGA cancer datasets. **G**. The mRNA level of PTP4A2 and miR-21 from Testicular Germ Cell Tumors (TGCT, n=156) was plotted and the Pearson rank correlation analyses were performed. Positive correlation coefficients suggest a positive correlation between PTP4A2 mRNA and miR-21 in the cancer samples (*****p*<0.0001). **H**. Patient samples from TGCT were divided into low expression and high expression groups based on the mean PTP4A2 mRNA level, and then the miR-21 level of the two groups was plotted. miR-21 level was significantly higher in the high PTP4A2 expression group than the low PTP4A2 expression group by Mann-Whitney tests, (****p*=0.0002). **I**. Patient samples from KIRC were divided into low expression and high expression groups based on the mean PTP4A2, PTP4A3 or miR-21 mRNA level. Patients with high PTP4A2 (***p*=0.0060), PTP4A3 (***p*=0.0078) or miR-21 (*****p*<0.0001) mRNA level have significantly reduced overall survival in KIRC by Kaplan-Meier survival analysis. **J**. Patient samples from LGG were divided into low expression and high expression groups based on the median PTP4A2, PTP4A3 or miR-21 mRNA level. Patients with high PTP4A2 (*****p*<0.0001), PTP4A3 (**p*=0.0339) or miR-21 (*****p*<0.0001) mRNA level have significantly reduced overall survival in LGG by Kaplan-Meier survival analysis.


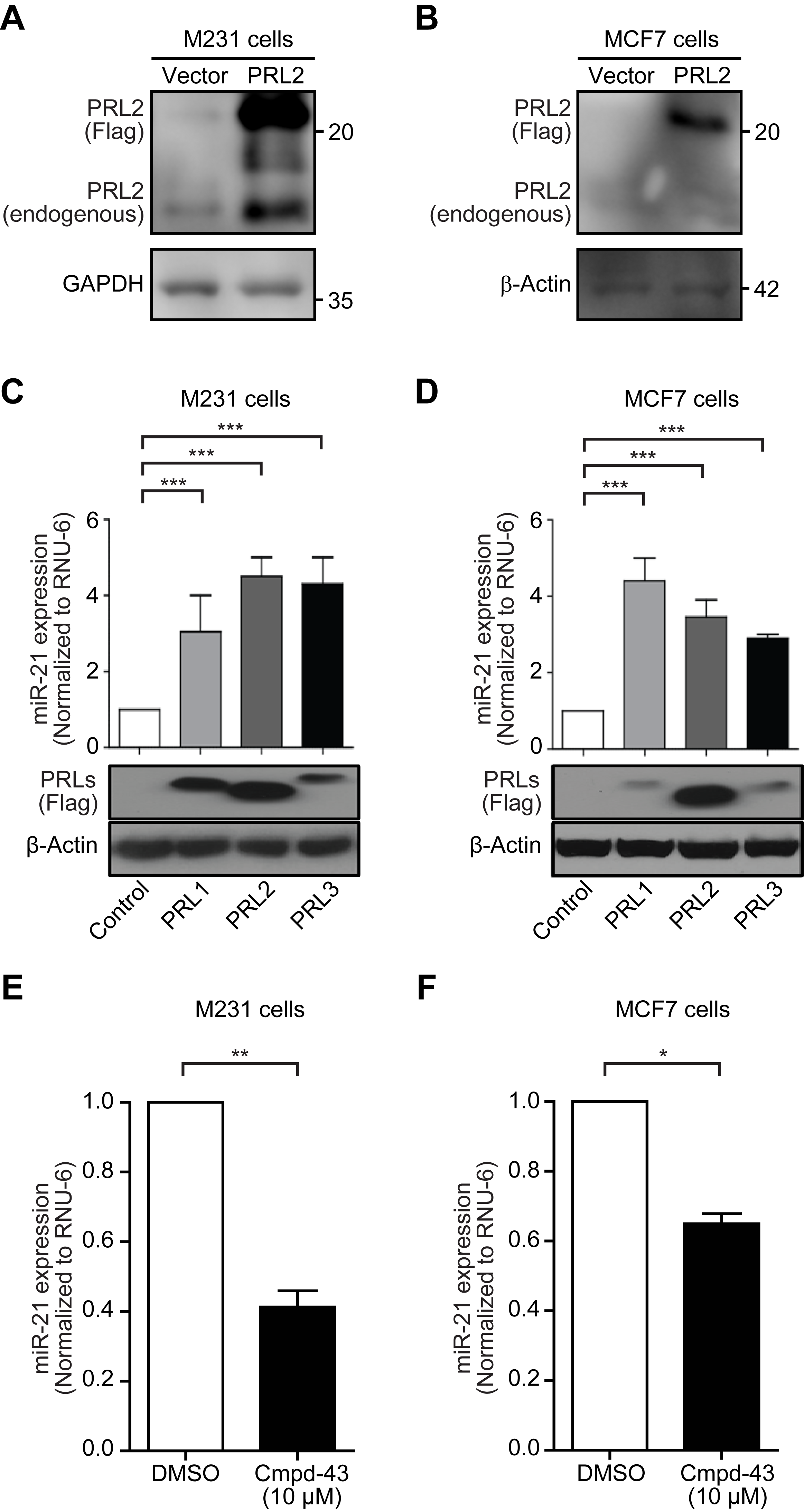


**Figure S2**. **PRL2 mediated miR-21 expression contributes to its oncogenic function.**

**A.** Western blotting showing relative PRL2 overexpression compared to endogenous PRL2 in control and PRL2 overexpressing M231 cells, detected by PRL2 and GAPDH antibodies. **B.** Western blotting showing relative PRL2 overexpression compared to endogenous PRL2 in control and PRL2 overexpressing MCF7 cells, detected by PRL2 and β-Actin antibodies. **C.** qRT-PCR analysis of miR-21 expression in M231 cells stably expressing control, PRL1, PRL2 and PRL3, normalized to RNU-6 snRNA. Each sample was analyzed in triplicate, and the Student t test was used to measure significance (***p<0.001). Western blotting showing PRLs protein expression in PRLs-Flag or control vector. **D.** qRT-PCR analysis of miR-21 expression in MCF7 cells stably expressing control, PRL1, PRL2 and PRL3, normalized to RNU-6 snRNA. Each sample was analyzed in triplicate, and the Student t test was used to measure significance (***p<0.001). Western blotting showing PRLs protein expression in PRLs-Flag or control vector. **E**. qRT-PCR analysis of miR-21 expression comparing DMSO control and Cmpd-43 treated (10 μM for 2 days) M231 cells, normalized to RNU-6 snRNA. Each sample was analyzed in triplicate, and the Student t test was used to measure significance (**p<0.01). **F**. qRT-PCR analysis of miR-21 expression comparing DMSO control and Cmpd-43 treated (10 μM for 2 days) MCF7 cells, normalized to RNU-6 snRNA. Each sample was analyzed in triplicate, and the Student t test was used to measure significance (*p<0.05).

**Figure S3**. **PRL2-mediated miR-21 upregulation is transcriptional regulated by STAT3.**


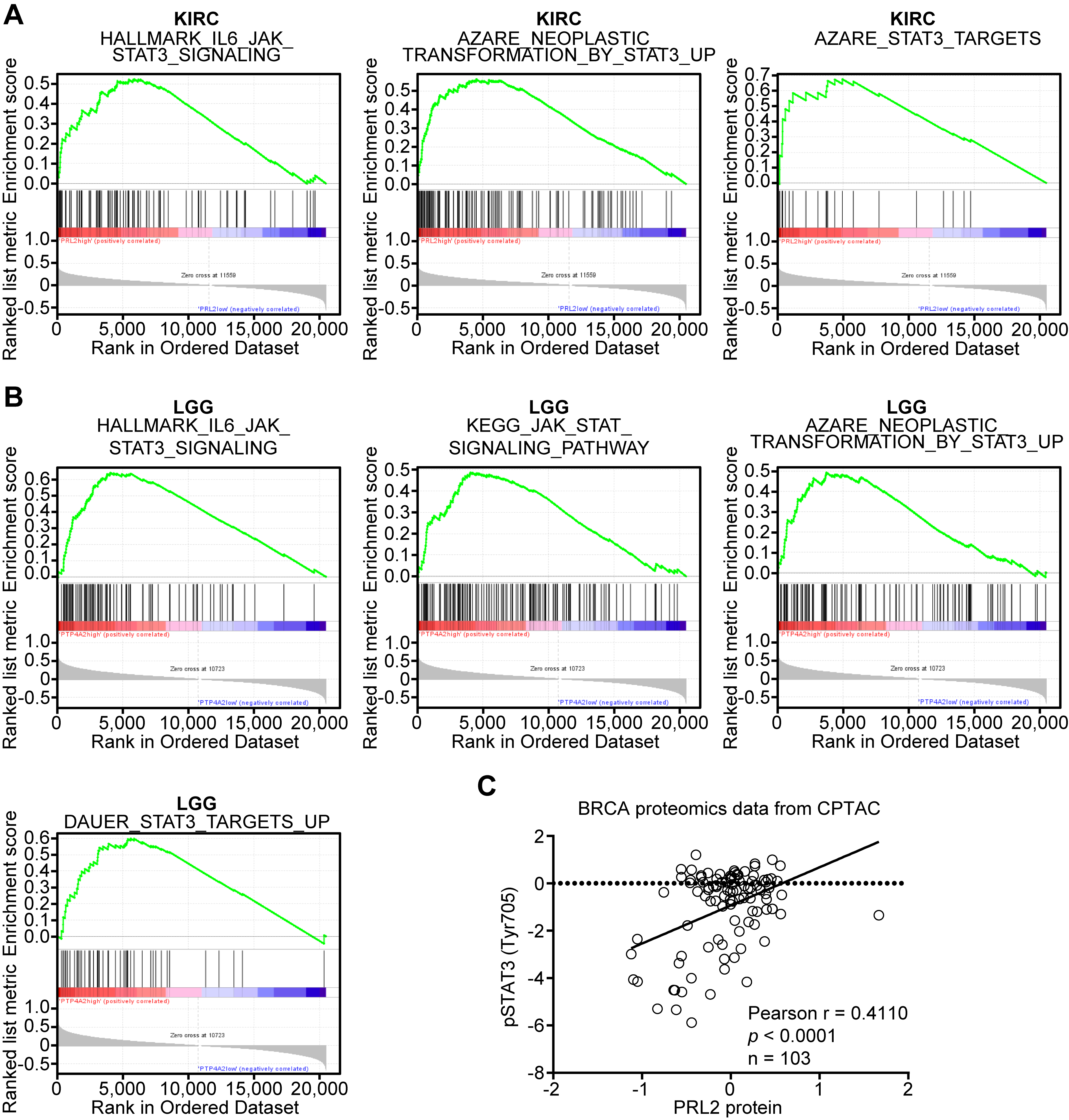


**A**. The enrichment score of Hallmark IL6–JAK–STAT3 pathway and other STAT3 related gene signature with high PRL2 expression in KIRC by GSEA analysis. **B**. The enrichment score of Hallmark IL6–JAK–STAT3 pathway and other STAT3 related gene signature with high PRL2 expression in LGG by GSEA analysis. **C**. Pearson correlation analysis of PRL2 protein level and pSTAT3 (Tyr705) level in TCGA BRCA patient samples by proteomics data from the Clinical Proteomic Tumor Analysis Consortium (CPTAC).

**Figure S4**. **PRL2 dephosphorylates JAK2 at tyrosine 570 to promote STAT3 activation.**


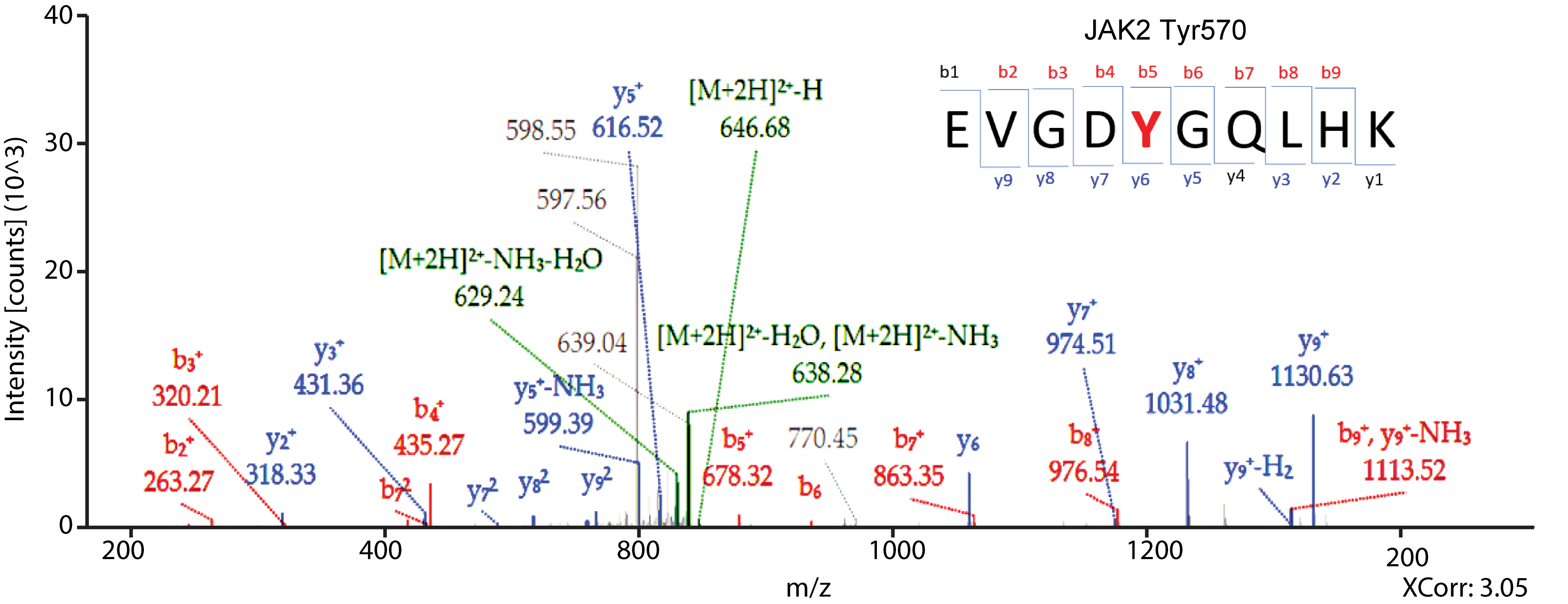


The spectrum of JAK2 Tyr570 containing peptide EVGDpYGQLHK in PRL2 WT and KO MEF cells identified by the LC-MS/MS.
